# Study of Hydroxypropyl β-Cyclodextrin and Puerarin Inclusion Complexes Encapsulated in Sodium Alginate-Grafted 2-Acrylamido-2-Methyl-1-Propane Sulfonic Acid Hydrogels for Oral Controlled Drug Delivery

**DOI:** 10.3390/gels9030246

**Published:** 2023-03-20

**Authors:** Abid Naeem, Chengqun Yu, Weifeng Zhu, Zhenzhong Zang, Yongmei Guan

**Affiliations:** Key Laboratory of Modern Preparation of Traditional Chinese Medicines, Ministry of Education, Jiangxi University of Chinese Medicine, Nanchang 330004, China; 20214003@jxutcm.edu.cn (A.N.); yuchengqun@jxutcm.edu.cn (C.Y.); 19930220@jxutcm.edu.cn (W.Z.)

**Keywords:** hydrogel, flavonoids, antioxidant, inclusion complexes, oral formulation

## Abstract

Puerarin has been reported to have anti-inflammatory, antioxidant, immunity enhancement, neuroprotective, cardioprotective, antitumor, and antimicrobial effects. However, due to its poor pharmacokinetic profile (low oral bioavailability, rapid systemic clearance, and short half-life) and physicochemical properties (e.g., low aqueous solubility and poor stability) its therapeutic efficacy is limited. The hydrophobic nature of puerarin makes it difficult to load into hydrogels. Hence, hydroxypropyl-β-cyclodextrin (HP-βCD)-puerarin inclusion complexes (*PIC*) were first prepared to enhance solubility and stability; then, they were incorporated into sodium alginate-grafted 2-acrylamido-2-methyl-1-propane sulfonic acid (SA-*g*-AMPS) hydrogels for controlled drug release in order to increase bioavailability. The puerarin inclusion complexes and hydrogels were evaluated via FTIR, TGA, SEM, XRD, and DSC. Swelling ratio and drug release were both highest at pH 1.2 (36.38% swelling ratio and 86.17% drug release) versus pH 7.4 (27.50% swelling ratio and 73.25% drug release) after 48 h. The hydrogels exhibited high porosity (85%) and biodegradability (10% in 1 week in phosphate buffer saline). In addition, the in vitro antioxidative activity (DPPH (71%), ABTS (75%), and antibacterial activity (*Staphylococcus aureus*, *Escherichia coli, and Pseudomonas aeruginosa)* indicated the puerarin inclusion complex-loaded hydrogels had antioxidative and antibacterial capabilities. This study provides a basis for the successful encapsulation of hydrophobic drugs inside hydrogels for controlled drug release and other purposes.

## 1. Introduction

Flavonoids are a group of chemical compounds found in plants. The phenolic compounds present in plants include flavonols, flavan-3-ols, anthocyanidins, flavones, flavanones, and isoflavones. There has been considerable scientific and therapeutic interest in the use of these substances found in fruits, vegetables, stems, grains, barks, roots, and flowers [1]. Flavonoids have played an important role in the treatment of many diseases since ancient times. Several flavonoids have been shown to have the functions of scavenging free radicals, improving antioxidant activity, displaying anti-inflammatory properties, possessing antitumor properties, possessing antithrombogenic properties, as well as possessing antibacterial properties [2,3].

Puerarin, a bioactive compound extracted from the roots of *Pueraria lobata* (Willd.) Ohwi, or Ge-gen in Chinese. Ge-gen has been used as a source of food, medicine, and fodder for more than 1000 years in ancient China, and it is one of the oldest medicinal herbs in history. Puerarin is one of three important isoflavonoids that possess anti-inflammatory, antioxidant, anticancer, anti-Parkinson’s disease, anti-Alzheimer’s disease, antiosteoporosis, cardioprotective, and neuroprotective properties [4]. Additionally, it can relieve postmenopausal symptoms, diabetic complications, endometriosis, alcohol-related problems, act as an anti-intoxication agent, exhibit protective effects against immunological damage, liver damage, and protect the mucosa of the stomach from damage caused by chronic stress [5]. Therefore, puerarin has the potential to be used in nutraceutical and pharmaceutical products. However, its low water solubility limits its use in pharmaceutical formulations.

According to the biopharmaceutics classification system, puerarin is categorized as an IV drug based on its low solubility and low intestinal permeability values. The solubility of puerarin in an aqueous solution is 0.46 mg/mL, its partition coefficient is 1.95, and its maximum solubility is 7.56 mg/mL at pH 7.4 (phosphate buffer) [6]. Therefore, it can be classified as an intravenous drug. Currently, puerarin is available only as an intravenous injection and eye drop due to its poor water-solubility and permeability. Furthermore, due to the short elimination half-life, frequent injections are necessary. Consequently, the development of a novel drug delivery system for puerarin is important. Many techniques have been investigated for improving its water solubility and stability, including enzymatic and chemical condensation, nanodispersion, encapsulation in natural and synthetic polymers, and others. The use of cyclodextrins (CDs) to form inclusion complexes provides another effective option for solving this problem [7].

Cyclodextrins (CDs) are cyclic oligosaccharides composed of glucopyranose units that are linked together by α-1,4 bonds, resulting in a cone-shaped structure. This cone-shaped structure enables CDs to enclose hydrophobic molecules and forms inclusion complexes. Inclusion compounds are prepared in such a way that positive modifications are made to the properties of guest molecules, including the enhancement of dissolution of insoluble substances, stabilization of nonstable materials against degradation by heat and light, masking of undesirable odors and tastes, control of drug release and fragrance-taste substances, and separation by chromatography [8]. Therefore, CDs and their inclusion complexes are a suitable delivery platform for many different types of products, including cosmetics, food, and pharmaceuticals. Natural CDs, or first-generation CDs, are classified as α-CD, β-CD, and γ-CD [9]. Each of these CDs contains 6, 7, and 8 glucopyranose units, respectively. β-CD is commonly used because it is inexpensive, readily available, and capable of forming complexes. However, due to its poor soluble nature, unmodified β-CD is limited in its application. Thus, modified forms of β-CDs have been synthesized and used, such as methylated-β-cyclodextrin (Me-β-CD) and (2-hydroxy) propyl-β-cyclodextrin (HP-βCD) [10]. Hydroxypropyl-β-cyclodextrin (HP-βCD) offers several advantages over other cyclodextrins, including water solubility, favorable animal tolerance, excellent absorption, high bioavailability, and excellent solubility. Therefore, it is frequently used as a solubilizer or as an excipient in pharmaceuticals. In addition, the Food and Drug Administration (FDA) has approved the use of HP-βCD as a solubilizer in various medications [11].

“Hydrogels” are three-dimensional (3D) structures formed by crosslinking physically or chemically, which can retain more water without losing structural integrity. The high water content of hydrogels has a significant impact on their permeability, surface properties, biocompatibility, and mechanical properties. Hydrogels can be manufactured using a variety of chemical processes; they can be prepared through three-dimensional polymerization by polymerizing hydrophilic monomers with crosslinking agents, or they can be prepared by polymerizing hydrophilic polymers directly [12]. The process of polymerization is usually triggered by compounds that contain free radicals or by the application of radiation, such as gamma, ultraviolet, or electron beams [13]. Hydrogels are extensively researched for their potential use in the development of medical devices and drug delivery systems due to their hydrophilicity, stability, swelling, gelling, biocompatibility, biodegradability, low toxicity, and solute control characteristics. Hydrogels maximize drug delivery benefits by minimizing the disadvantages of conventional delivery systems [14].

Sodium alginate is one of the most commonly found macromolecules in nature that is composed of two units, namely poly-β-1, 4-D-mannuronic acid (M units) and α-1, 4-ʟ-glucuronic acid (G units), in different proportions through 1–4 linkages. Sodium alginate can be obtained from marine algae or can be produced by bacteria. Sodium alginate is an excellent material for the preparation of hydrogels due to its abundance, low cost, nontoxic characteristics, water-soluble properties, biodegradability, and biocompatibility [15]. Sodium alginate has been used in cosmetics, food, and pharmaceutical preparations for over 100 years [16,17].

2-acrylamido-2-methylpropanesulfonic acid (AMPS) is a hydrophilic ionic monomer that plays a key role in the preparation of hydrogels for the delivery of drugs. AMPS-based hydrogels exhibit pH-independent swelling behavior due to the presence of ionizable sulfonic groups. Furthermore, increasing the concentration of AMPS increases the swelling index of hydrogels as a result of the increase in ionic groups influx into the hydrogels [18]. Crosslinking of hydrogels was achieved with the use of ethylene glycol dimethacrylate (EGDMA) [19].

In this study, super-swellable (SA-g-AMPS) hydrogels are developed that can absorb significant amounts of water, maintain their structural integrity, and are highly porous and biodegradable. So, it can be used for delivering many different types of hydrophilic/hydrophobic drugs. These hydrogels are capable of carrying higher drug loads and releasing them in a controlled manner. Additionally, we have shown that hydrophobic drugs (puerarin) can also be incorporated into hydrogels by forming inclusion complexes (PIC). This approach can be used to stabilize hydrophobic drugs or light-sensitive or pH-sensitive compounds, which can then be loaded into hydrogels, which can release them in a controlled manner without compromising their stability. Based on the above considerations, the purpose of this study was to develop a hydrogel based on polysaccharide sodium alginate and hydrophilic monomer 2-acrylamido-2-methylpropanesulfonic acid (AMPS) and loaded with hydrophobic drug (puerarin) inclusion complexes with 2-hydroxypropyl-β-cyclodextrin (*PIC*) so that puerarin can be released for a prolonged period of time to improve its solubility, stability, bioavailability and reduce its dosing frequency. Fourier transform infrared spectroscopy (FTIR), X-ray diffraction (XRD), differential scanning calorimetry (DSC), thermogravimetric analysis (TGA), the porosity of the hydrogel network, the volume fraction of polymer (V2,s), sol-gel fraction analysis, crosslinking molecular weight (Mc), and biodegradation studies were used to characterize the hydrogel’s structure. The swelling and release behavior of hydrogels was also examined in different pH media (pH 1.2 and pH 7.4) by using different concentrations of polymer and crosslinker. Moreover, hydrogels containing puerarin inclusion complexes were evaluated for their antioxidant activity (DPPH and ABTS assays) and antibacterial activity (*Staphylococcus aureus*, *Pseudomonas aeruginosa*, and *Escherichia coli*).

## 2. Results and Discussion

### 2.1. ^1^H NMR and FTIR Analysis

**Figure 1:**
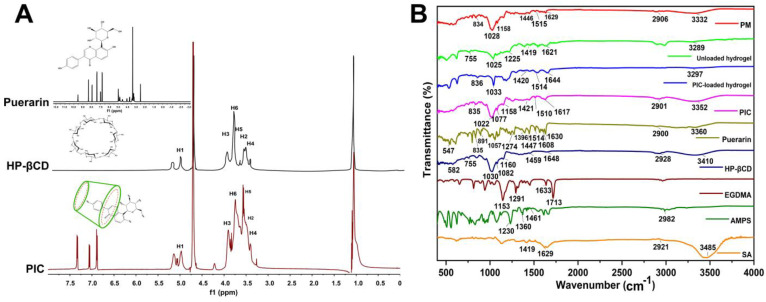
^1^H NMR spectra of puerarin and HP-βCD inclusion complexes (**A**), and FTIR spectra of pure components and hydrogels (**B**).

^1^H NMR analysis confirmed the incorporation of the guest (puerarin) into the host (HP-βCD hydrophobic cavity). A change in chemical shift values (Δδ) occurs when puerarin inclusions occur in HP-βCD cavities, resulting in a change in the chemical and electronic environment of protons. This chemical shift provides information regarding which part of a guest molecule has been inserted into the CD cavity. The ^1^H NMR spectra of HP-βCD, puerarin, and their inclusion complexes are shown in Figure 1A. The ^1^H chemical shifts of free HP-βCD and puerarin were consistent with previous studies [20,21]. It shows that the inclusion complexation of puerarin with HP-βCD has a negligible effect on the values of the H-2 and H-6 protons of HP-βCD (0.008 ppm, 0.007 ppm). When puerarin was added to the HP-βCD solution, the H-4 on the inner surface of HP-βCD were shielded, and H-1, H-3, and H-5 were deshielded. The shielding was due to the ring current effect of puerarin’s aromatic system, which indicated that puerarin had entered into the HP-βCD cavity.

The FTIR spectra of sodium alginate, AMPS, puerarin, EGDMA, PIC, HP-βCD, and unloaded and PIC-loaded hydrogels are shown in Figure 1B. The FTIR spectrum of sodium alginate showed absorption bands at 3485 cm^−1^ (OH stretching), 1629 cm^−1^ (COO−asymmetric stretching), and 1419 cm^−1^ (COO− symmetric stretching), which are in line with previous studies [22]. The FTIR spectra of AMPS showed vibration bands at 1461 cm^−1^ corresponding to the binding vibration of CH_2_. The vibration band at 1360 represents -C-O stretching vibration; the characteristic bands present at 2982 cm^−1^ suggested –CH stretching of –CH_2_. The bands at 1360 cm^−1^ are attributed to asymmetric stretching of the S=O group, while 1230 cm^−1^ represents symmetric stretching of the S=O group [23]. The FTIR spectra of EGDMA showed a vibration band at 1713 cm^−1^, which corresponds to the stretching vibrations of C=O, and bands at 1633, 1291, and 1153 cm^−1^ are ascribed to the C=C and C–O stretching vibrations of ester groups [24,25]. The FTIR spectrum of pure HP-βCD demonstrated the stretching vibration of –OH at 3410 cm^−1^ and a symmetric stretching vibration of C-H at 2928 cm^−1^. The characteristic bands present at 1160 cm^−1^ and 1030 cm^−1^ were attributed to C-O stretching vibrations. This is consistent with results reported by other researchers [26]. The FTIR spectrum of puerarin revealed the prominent absorption bands of hydroxyl groups (3360 cm^−1^), the aromatic conjugated carbonyl groups (1630 cm^−1^), and the aromatic nuclei (1608, 1514, and 1447 cm^−1^). This is consistent with the findings of others [27]. In the spectrum of the physical mixture (PM), characteristic bands, such as the stretching vibration of the hydroxyl groups at 3332 cm^−1^, carbonyl groups at 1629 cm^−1^, and vibrations of benzene rings at 1515 and 1446 cm^−1^, could be observed. Furthermore, the bands of hydroxyl groups were broader and partially overlapped with the bands at 2906 cm^−1^. This suggested that there was no or minor interaction between puerarin and HP-βCD in the physical mixture [28]. The FTIR spectrum of PIC showed vibration bands at 3352 cm^−1^ corresponding to –OH vibration, and the band at 1022 cm^−1^ corresponds to C-O stretching vibration. The bands at 1617 cm^−1^ and 1510 cm^−1^ belong to the aromatic nuclei of puerarin, and the presence of various other bands suggests the inclusion of puerarin in HP-βCD cavity. The FTIR spectra of the PIC-loaded hydrogels showed a different spectrum when compared with its parent components. The bands appearing at 1514 cm^−1^ belong to the bands of the aromatic nucleus of puerarin, and the bands at 1033 cm^−1^ correspond to the C-O stretching vibration of HP-βCD. The vibration band at 1420 cm^−1^ is attributed to sodium alginate (COO− symmetric stretching). The FTIR spectrum of the blank hydrogel showed absorption bands at 3289 cm^−1^ (OH stretching). The vibration band at 1420 cm^−1^ is attributed to sodium alginate (COO− symmetric stretching). The FTIR spectra of the synthesized hydrogel showed some new peaks and little shifting, overlapping, and disappearance of some peaks (characteristic peaks of pure components) within polymeric networks. Therefore, a new grafted polymer network and successful entrapment of the drug within the hydrogel structures were achieved [29].

### 2.2. TGA Study

**Figure 2:**
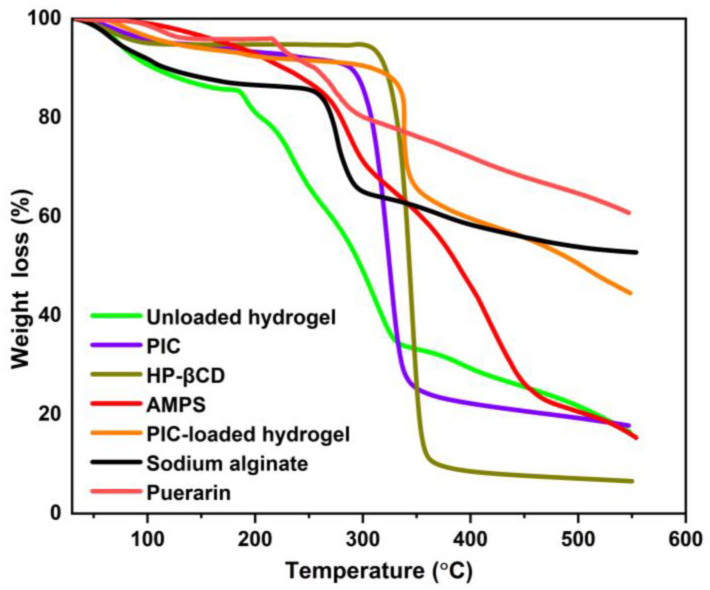
TGA of HP-βCD, puerarin, sodium alginate, AMPS, *PIC*, unloaded and *PIC*-loaded hydrogels.

TGA was performed to evaluate the thermal stability of the polymers, the inclusion complexes, and the developed hydrogels (Figure 2). The TGA results of AMPS indicated a weight loss of 6% when the temperature reached 208 °C; further weight loss of 20% was observed within the temperature range of 210 °C to 250 °C, indicating moisture and water loss. The sulfonic acid group also began to decompose between 250 and 340 °C, resulting in an additional 20% weight loss [30]. The weight loss observed in the HP-βCD samples below 80 °C was attributed to water evaporation, and the weight loss observed between 305 °C and 380 °C was due to HP-βCD degradation, which is consistent with other studies [31]. In pure sodium alginate, the first loss of weight before 100 °C can be attributed to the loss of absorbed water. The rapid weight loss at temperatures between 200 °C and 270 °C can be attributed to the decomposition of sodium alginate as the temperature increases [32]. The PIC samples showed water loss from 40 °C to 280 °C. When the temperature was between 280 °C and 330 °C, the weight loss could be attributed to PIC degradation.

The unloaded hydrogel developed initially lost 18% of its weight due to dehydration in the temperature range of 30 to 180 °C, followed by a 46% weight loss at 180 to 320 °C due to the breaking of polymer bonds. The decomposition of polymeric networks began after 320 °C and continued until the complete degradation of the polymer backbone occurred. In comparison to the reactants, the hydrogels with increased residual weight showed enhanced stability against thermal degradation over the entire temperature range studied. Further, because of the enhanced strength and interaction between the polymer and monomer, the degradation of the developed hydrogel begins at elevated temperatures and at a slower rate than the reactions of its individual components. Thermal stability has been enhanced with the shifting of endothermic peaks to elevated temperatures, which indicates the formation of rigid networks that are more stable [33].

### 2.3. DSC Analysis

**Figure 3:**
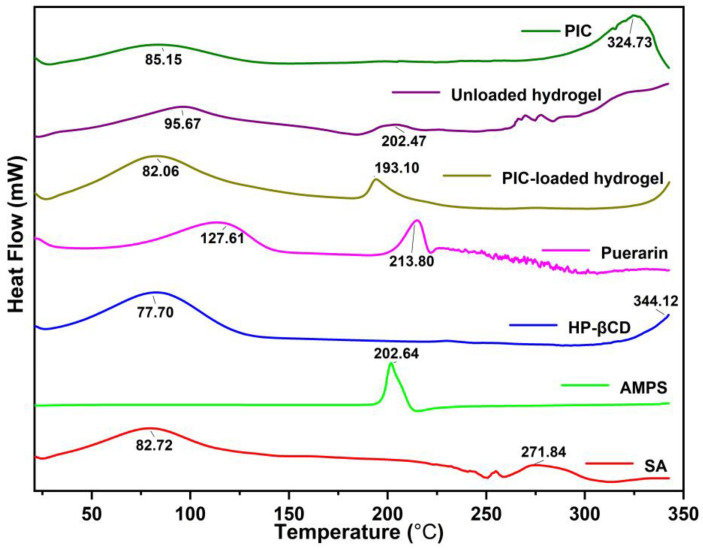
The DSC of puerarin, HP-βCD, SA, *PIC*, AMPS, unloaded, and *PIC*-loaded hydrogels.

The DSC analysis results are shown in Figure 3. AMPS showed a sharp endothermic peak at 202 °C, indicating the decomposition of the sulphonic acid groups [34]. HP-βCD showed water loss at 77.70 °C and a melting peak around 344.12 °C. The DSC thermogram of sodium alginate exhibited an endothermic peak (90 to 100 °C) corresponding to the heat of evaporation of the associated water of hydration, while the other peaks signified the heat required for the dissociation of intra-and intermolecular hydrogen bonding within the polymeric matrix and polymeric degradation [35]. The sodium molecules undergo pyrolysis between 220 and 271 °C, resulting in a maximum rate of decomposition [36]. The endothermic peak of puerarin appeared at 213.80 °C, corresponding to the melting point, and a broad peak appeared at 127.61 °C, which may correspond to the polycrystalline form of the drug. This is consistent with the findings of others [37]. In contrast, the thermal spectra of PIC mainly showed the characteristics of an HP-βCD curve, and the endothermic peak of puerarin completely disappeared, indicating the formation of an associative structural molecular complexation [38]. The hydrogels exhibited an exothermic peak at around 200 °C, which indicates the presence of AMPS in the formulation. The newly developed structure results in a formulation that is thermally stable.

### 2.4. XRD Study

**Figure 4:**
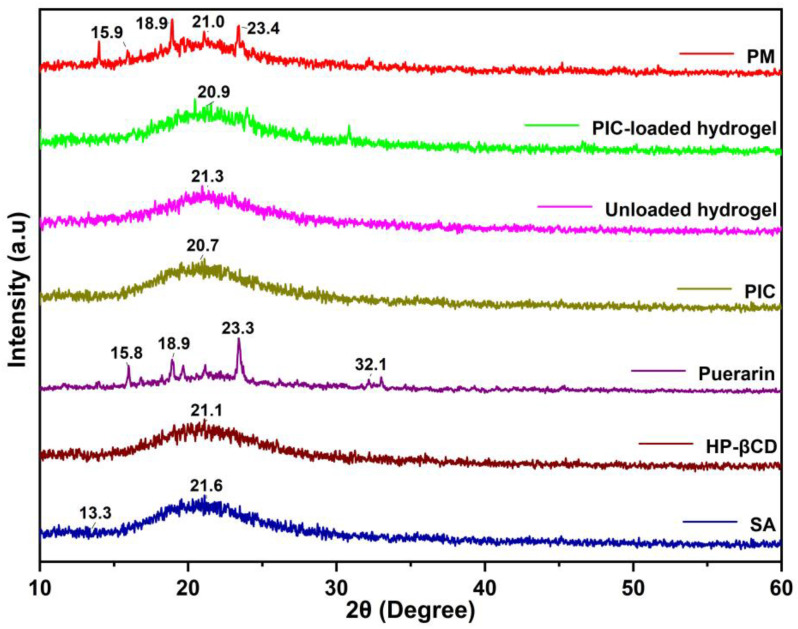
The XRD pattern of the formulation ingredients, inclusion complexes, and hydrogels synthesized.

The XRD spectra of all the polymers and fabricated hydrogels are shown in Figure 4. The HP-βCD sample shows a broad peak at 2θ = 21.1°, which suggests that the HP-βCD is in an amorphous state. This is similar to the results of other studies [31]. The sodium alginate spectra indicated two reflections at 2θ = 21.6° and 2θ = 13.3°, which is consistent with the results of other researchers [39]. The XRD pattern of puerarin showed several different diffraction peaks, such as 2θ = 15.8°, 18.9°, 23.3°, and 32.1°, indicating its crystalline properties, and these results are consistent with those of others [40]. XRD analysis of the PIC did not reveal any crystallization peaks, showing that puerarin was encapsulated within HP-βCD to form inclusion complexes. The physical mixture (PM) gave the superimposed patterns of crystalline puerarin and amorphous HP-βCD. The unloaded hydrogel diffractogram shows that there is a broad peak at 2θ = 20.9°. However, the diffractogram of the drug-loaded hydrogels shows two broader peaks at 2θ = 21.3° and 30.9°, whereas no other intense peak of the drug was displayed at its respective region, which might be due to the physical interaction of the drug with polymeric blends, which interfered with the purity of the drug, and hence, the crystal lattice characteristic of the drug was reduced.

### 2.5. SEM

**Figure 5:**
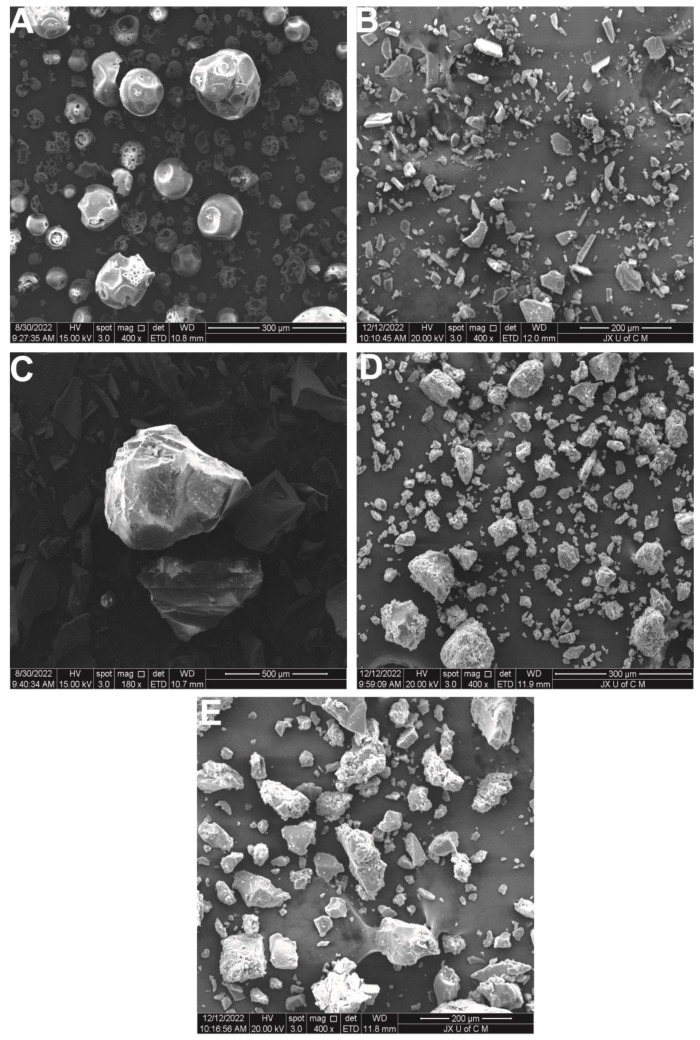
SEM images of HP-βCD (**A**), puerarin (**B**), *PIC* (**C**), unloaded hydrogels at 400× (**D**), and *PIC*-loaded hydrogels at 400× (**E**).

The SEM images of HP-βCD show a spherical and cavity-filled porous structure [41]. The puerarin structure is asymmetrical and bulky. An inclusion complex with puerarin altered the spherical shape of HP-βCD. The inclusion resulted in a distinct solid phase with a different morphology. This is consistent with the XRD results. Figure 5 shows the dense, irregular, and rough surface of the hydrogels [42]. As hydrogels dry, the polymeric networks can collapse, leaving rough surfaces. The hydrogels feature a high concentration of crosslinked polymeric chains, which allows them to trap drugs through their pores. Swelling can occur when media penetrates the pores, causing the release of drugs. Smooth surfaces and solid masses in hydrogels are key to the stabilization of polymeric systems.

### 2.6. Mechanical Properties of Hydrogels

Hydrogels must be evaluated for their mechanical properties prior to being used as drug delivery systems. These include their tensile strength (TS) as well as their elongation of break (EAB). It has been found that an increase in EGDMA is associated with an increase in tensile force (Table 1) [43]. AMPS can be described as an anionic monomer, and the increase in AMPS content may result in a decrease in the mechanical strength of the hydrogel. It could be caused by an increase in osmotic and electrostatic pressures. Sodium alginate is a naturally occurring polysaccharide derived from brown algae. The mechanical strength and tensile strength of hydrogels increase with an increasing amount of sodium alginate. This behavior may be explained by the increased crosslinking density caused by sodium alginate. The mechanical properties of these hydrogels are similar to those observed by other researchers [44].

### 2.7. Sol–Gel Evaluation

During the polymerization reaction, the monomer, crosslinker, and polymer react to produce a crosslinked polymeric hydrogel network. However, some of the hydrogel may remain uncrosslinked, which is termed the “sol fraction”, while the crosslinked portions are called the “gel portion”. The sol portion does not undergo crosslinking due to the inability to find reactive sites during polymerization. All hydrogel formulations have been analyzed using the Sol-gel method. Hydrogels can be classified according to their degree of crosslinking or uncrosslinking based on this technique. Sol-gel analysis is generally used to determine the percentage of polymers that are uncrosslinked [45]. The gel fraction is increased by AMPS and hydrophilic monomers. EGDMA is a crosslinking ingredient that can trigger the formation of the gel. The gel fraction increases as the content of EGDMA increases. The results showed a rise in gel fraction as the composition of sodium alginate increased while the sol fraction decreased and vice versa. The gel fraction increases when sodium alginate has more space to react chemically [46].

### 2.8. Porosity Assessment

**Figure 6:**
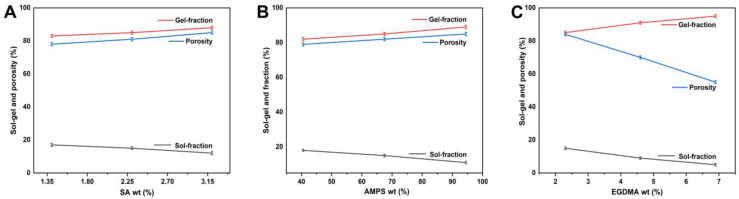
Effect of sodium alginate (**A**), AMPS (**B**), and EGDMA (**C**) on sol–gel fraction and porosity of fabricated hydrogels.

Porosity plays a significant role in the swelling, loading, and release of drugs from hydrogels [47]. There is a direct relationship between the size of the pores and the amount of fluid absorbed. As a result, drug loading, release, and swelling are increased. As EGDMA concentration increases, porosity decreases due to tight junctions and crosslinked bulk densities that hinder the flexibility of the system (Figure 6A–C). The porosity of a material may increase with an increase in AMPS concentration. This is because the sulfonate group of AMPS can generate greater electrostatic forces. A hydrophobic alkyl group in AMPS reduces hydrogen bond interactions by forming microregions with hydrophobic properties. Studies have shown that hydrogel preparations contain a greater number of pores and networks than conventional gels. Several studies have shown that the porosity increases as sodium-alginate concentration increases. This is due to the formation of interconnected pores following the incorporation of sodium alginate into the hydrogel [48].

### 2.9. Biodegradation Determination

**Figure 7:**
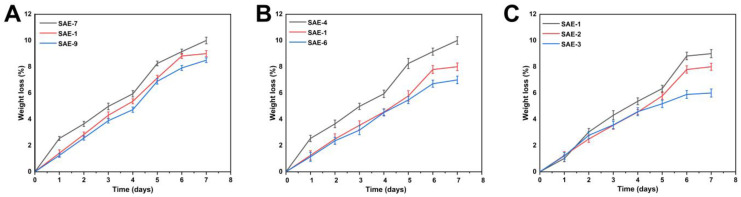
Effect of ingredients on the biodegradation of hydrogels: sodium alginate (SAE-7,1,9) (**A**), AMPS (SAE-4,1,6) (**B**), and EGDMA (SAE-1,2,3) (**C**) on the in vitro biodegradation of the developed hydrogels.

The biodegradation study was carried out to determine the rate at which the prepared hydrogel degraded, as shown in Figure 7A–C. The hydrogel’s degradation rate is significantly affected by the material-weight ratio [49]. The degradation rate of the hydrogels developed with EGDMA decreased as a result of an increase in EGDMA concentration. This could be due to the formation of functional groups, which leads to large amounts of free radicals. These free radicals are important in the polymerization reaction. They can increase gel strength through increased crosslinking, which, in turn, reduces the degradation rate. Alginate has been extensively studied and is used in many biomedical applications because of its biocompatibility and biodegradability. It has a significant impact on the mechanical properties of the hydrogels, stabilizes the hydrogel network, and slows down their degradation.

### 2.10. Structural Parameters Analysis of SA-g-AMPS Hydrogels

The synthesized hydrogels were characterized by various structural parameters, including the average molecular mass between crosslinks Mc (degree of crosslinking), the volume fraction of the polymer V2,s (amount of fluid absorbed into the network), the solvent interaction parameter (χ), number of repeating units (crosslinks N), and diffusion coefficient (D) [50]. The values for various structural parameters are listed in Table 2. These parameters play a critical role in determining the compatibility of hydrogels with their solvent and their maximum absorbency or holding capacity. V2, S, and χ increased as EGDMA levels increased. This is consistent with the findings of other researchers. Similar results were observed for Mc and N. This is due to an increase in crosslinking density in conjunction with an increase in EGDMA.

### 2.11. Swelling Study

**Figure 8:**
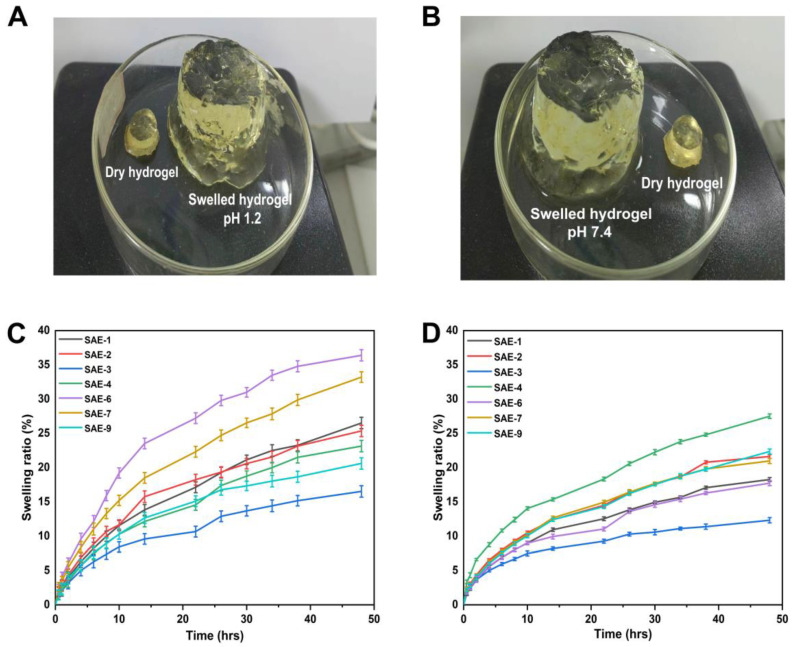
The appearance of synthesized hydrogels at pH 1.2 (**A**) and pH 7.4 (**B**). Hydrogel swelling curves over time at pH 1.2 (**C**) and 7.4 (**D**).

The hydrogels were formulated utilizing variable amounts of polymer (sodium alginate), monomer (AMPS), and crosslinker (EGDMA) to investigate their effects on the swelling of the hydrogels in both acidic (pH 1.2) and basic (pH 7.4) conditions [51]. Hydrogels expand when placed in water or buffer media due to the hydrophilicity of the polymers and monomers. Figure 8 illustrates the swelling rate of a hydrogel at various pH levels over time. The results indicated that the swelling of the hydrogels was slightly higher at pH = 1.2 and slightly lower at pH = 7.4. This behavior may be caused by the protonation process occurring within AMPS. The swelling of the hydrogels increased with an increase in AMPS concentration. This may be due to the large amount of –CONH_2_ and –SO_3_OH groups present in AMPS, which, upon ionization, combine with water molecules, resulting in the swelling of hydrogels [52]. The increased swelling can be attributed to the ionization of the hydroxyl (–OH) functional groups of the hydrogels in the buffer media [53]. In acidic environments (pH 1.2), the hydrogen bonds between carboxylate groups contribute to polymer–polymer interactions and predominate over polymer–water interactions. This reduces the electrostatic repulsion between these groups, thereby favoring shrinkage, resulting in a relatively low swelling rate for the hydrogel. As the pH of the external swelling medium rises (pH 7.4), the carboxylic acid group within the SA molecule ionizes and produces -COO- ions, thereby causing the hydrogel to swell [54]. However, the swelling ratio of the hydrogel decreased as the amount of alginate increased, which may be associated with the formation of more dense and tight networks. A decrease in swelling degree was observed with an increase in EGDMA concentration. The porosity of hydrogels decreases with increasing EGDMA concentration because the crosslinking density of hydrogels increases. Consequently, the water penetration into the hydrogel network is reduced, and swelling decreases as EGDMA concentration increases and vice versa.

### 2.12. Drug Release and Kinetic Data Modelling

**Figure 9:**
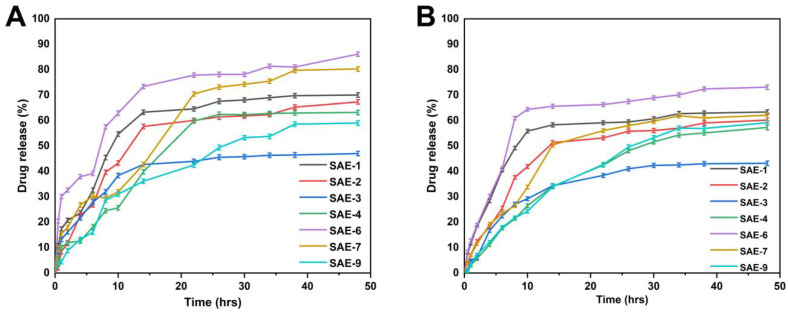
In vitro drug release of SA-g-AMPS hydrogels at pH 1.2 (**A**) and 7.4 (**B**).

Drug release from the fabricated hydrogels does not seem to be significantly affected by pH changes in the dissolution media, confirming pH-independent drug release. A possible explanation for the phenomenon can be found by taking into account the pka of AMPS (pka = ~1.9) [55]. It can be stated that normally, systems become ionized as the pH of the systems increases and its pka increases. Consequently, because AMPS has a pka = 1.9, it dissociates at a lower pH, generating an increased number of ionized -SO_3_ groups, which makes the system more ionic at a lower pH, causing an increase in electrostatic repulsion among the negatively charged -SO_3_ groups, leading to increased hydrogel swelling. Therefore, the hydrogels showed a higher release at a lower pH because a higher concentration of AMPS was used in the preparation of the hydrogels. In general, the larger the swelling of the hydrogel, the greater the rate at which the drug will be released. The concentration of the polymers, monomers, and crosslinkers influences the release of drugs, as opposed to pH-independency. Increased sodium alginate concentration may have caused a decrease in drug release as a result of the increased swelling of the polymeric networks, leading to the formation of a more viscous and thicker diffusional barrier, which can inhibit drug molecules from leaching from the system, thus preventing drug release [56]. Figure 9A shows that the amount of drug released varies from 49.15% to 86.17% at pH 1.2. SAE-6 had the highest drug release rate at pH = 1.2 (86.17%), while SAE-3 had the lowest drug release rate at pH = 1.2 (49.15%). The drug release rate was 43.25~73.25% at pH 7.4 (Figure 9B). SAE-6 had the highest drug release rate (73.25%), while SAE-3 had the lowest drug release rate (43.25%) at pH 7.4. The drug release curve revealed that the drug was released in a different manner at different pH levels. The drug release rate was slightly higher at pH 1.2 than at pH 7.4.

The hydrogel discs immersed in water/buffer media allow water molecules to diffuse into polymer networks as a result of an osmotic pressure gradient. Water diffusion causes the hydrogel discs to swell, resulting in the opening of channels, thereby allowing the drug to be released. A regression coefficient value of close to 1 was considered the most appropriate model to fit the release data. In Table S1, the values of the regression coefficients (r) are presented for the samples with different concentrations of sodium alginate (SAE-1, SAE-7, and SAE-9). The regression coefficient values of these samples were greater than zero-order and first-order, indicating that they followed Higuchi release kinetics. Based on the close values of the regression coefficient to 1, the samples with varying concentrations of the crosslinker EGDMA (SAE-1, SAE-2, and SAE-3) also followed Higuchi release kinetics. Additionally, the regression coefficient (r) values obtained for the Higuchi model of the samples with varying concentrations of AMPS (SAE-1, SAE-4, and SAE-6) suggest that the drug release mechanism is diffusion-controlled. All drug-loaded samples (SAE-1, SAE-2, SAE-3, SAE-4, SAE-6, SAE-7, and SAE-9) analyzed using the Korsmeyer-Peppas model showed non-Fickian diffusion exponents (n) [57].

### 2.13. Antioxidation Effects

**Figure 10:**
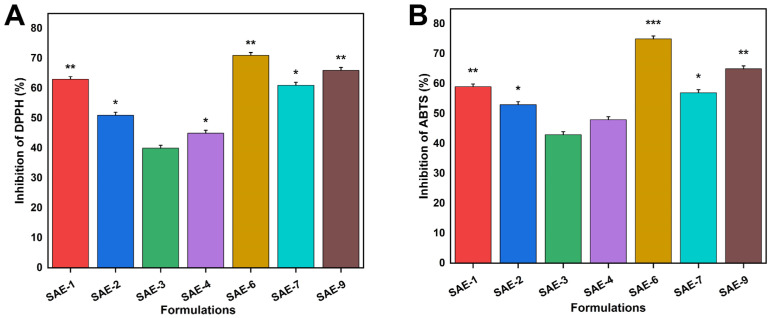
Antioxidation effect of SA-g-AMPS hydrogels against DPPH (**A**) and ABTS (**B**). Here, * shows the *p* value < 0.05, ** *p* < 0.01, and *** *p* < 0.001.

The antioxidant activity of the hydrogels was assessed by measuring their scavenging abilities against DPPH (Figure 10A) and ABTS (Figure 10B). The antioxidant activity of the four formulations (SAE-1 to SAE-2, SAE-6 to SAE-7, and SAE-7) was significantly higher than the other formulations. Sodium alginate, a natural polysaccharide that contains abundant hydroxyl- and carboxyl groups, is a common drug adjuvant that has antioxidant and immunomodulatory properties. The antioxidant performance increased with a gradual increase in the concentration of sodium alginate. Puerarin, a natural flavonoid, has a range of pharmacological properties, including anti-inflammatory and antibiotic effects. Puerarin has strong antioxidant properties. Puerarin, for example, inhibits lipid peroxidation by reducing superoxide anion production. Researchers used dextran sulfate sodium-induced colitis mouse models in order to demonstrate the antioxidant mechanism. The study results showed strong antioxidant properties in hydrogel-coated puerarin in various proportions [58].

### 2.14. Antibacterial Evaluation

**Figure 11:**
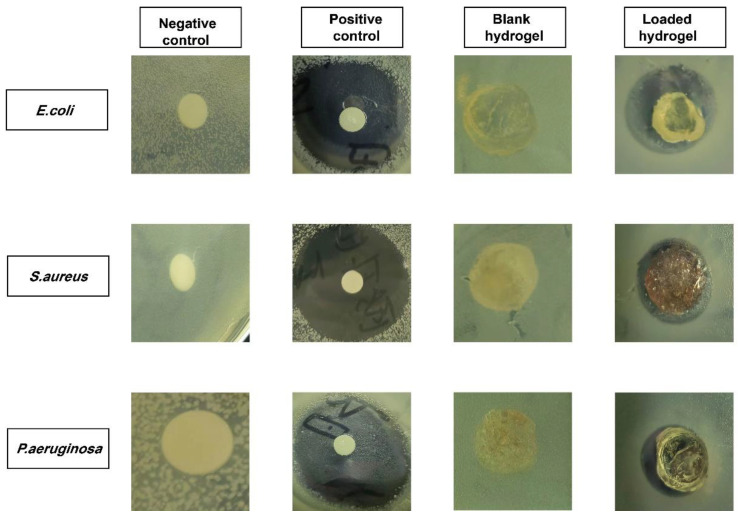
Antibacterial effects (zone of inhibition) of SA-g-AMPS hydrogels.

Antibacterial activities were determined against Gram-positive and Gram-negative bacteria, and their zones of inhibition are depicted in Figure 11. The negative control group and the blank hydrogel membrane group did not form zones, while the positive control group, i.e., 27 mm, 29 mm, and 23 mm, and PIC-loaded hydrogel discs, i.e., 15 mm, 9 mm, and 8 mm, showed clear zones against *E. coli*, *S. aureus*, and *P. aeruginosa*, respectively. The antibiotic cefepime has broad-spectrum activity and is effective against both Gram-negative and Gram-positive bacteria [59]. According to the results, cefepime exhibited a lower antibacterial effect against Gram-negative bacteria than against Gram-positive bacteria. The reason for this can be attributed to the structure of the bacteria’s cell wall. The cell wall of Gram-negative bacteria consists of three layers, namely, the outer membrane surrounded by peptidoglycans and the inner membrane surrounded by cytoplasm. In Gram-positive bacteria, the cell wall is thick, but it lacks the outermost membrane that is found in Gram-negative bacteria. It acts as a protective layer for Gram-negative bacteria, providing a barrier to the external environment. As a result, Gram-positive bacteria produce a larger zone of inhibition than Gram-negative bacteria.

## 3. Conclusions

In this study, puerarin inclusion complexes with HP-βCD were prepared to enhance the aqueous solubility of the drug. *PIC* showed an entrapment efficiency (EE%) of 85.50%, a drug loading (DL%) of 19%, and a yield of 90%, which is consistent with successful inclusion complexation. In the next step, sodium alginate-grafted AMPS controlled-release hydrogels were fabricated using a free radical polymerization method. FTIR, TGA, XRD, and DSC analyses were performed in order to verify the development of the *PIC* and the hydrogel network. In addition, the tests showed the successful loading of the *PIC* into the hydrogel. The porous nature of the hydrogel was confirmed by SEM analysis. A slightly higher swelling was observed in the hydrogels after 48 h at pH 1.2 (36.38% swelling) than at pH 7.4 (27.50% swelling). The synthesized hydrogels released drugs slightly more efficiently at pH 1.2 (86.17%) than at pH 7.4 (73.25%), with non-Fickian diffusion being the predominant mechanism of drug release. The release of the drug was prolonged, and the mechanical properties were improved with a higher polymer ratio and monomer concentration. Additionally, the hydrogels were found to be highly porous (85% porosity) and biodegradable (10% weight loss in one week). The developed hydrogels also demonstrated significant antioxidant activity in DPPH assays (inhibition of 71%), as well as in ABTS assays (inhibition of 75%). Furthermore, the hydrogels displayed excellent antibacterial properties against Gram-positive bacteria, such as *E. coli* and *S. aureus* (zones of inhibition of 15 and 9 mm, respectively), as well as Gram-negative bacteria, such as *P. aeruginosa* (zones of inhibition of 8 mm). In conclusion, HP-βCD-puerarin inclusion complexes loaded into sodium alginate-grafted AMPS hydrogels present an opportunity to deliver hydrophobic (which is difficult to load within hydrogels) or hydrophilic drugs for extended periods of time.

## 4. Materials and Methods

### 4.1. Materials

Sodium alginate from brown algae (MW: 216.12 g/moL, Purity ≥ 98), Hydroxypropyl beta-cyclodextrin (HP-βCD; MW: 1541.5 g/moL), 2,2-diphenyl-1-picrylhydrazyl (DPPH), 2,2′-azino-bis (3-ethylbenzothiazoline-6-sulfonic acid (ABTS), and Cefepime HCL were acquired from meilune biological company (Dalian, China). 2-Acrylamido -2-methyl-1-propanesulfonic acid (AMPS; MW: 207.25 g/moL), ethylene glycol dimethacrylate (EGDMA; MW: 198.22 g/moL), and ammonium persulfate (APS) were procured from Sigma-Aldrich (Saint Louis, MO, USA). Sodium bisulfite (SHS) was obtained from Shanghai Aladdin biochemical technology (Shanghai, China).

Bacterial strains, such as *Staphylococcus aureus* (*S. aureus*: ATCC25923HBJZ005), *Pseudomonas aeruginosa* (*P. aeruginosa*: ATCC27853HBJZ017), and *Escherichia coli* (*E. coli*: ATCC25922HBJZ087), were procured from Qingdao Hope Biotechnology, Co., Ltd. (Qingdao, China).

### 4.2. Synthesis of HP-βCD-Puerarin Inclusion Complexes (PIC)

HP-βCD-puerarin ICs were prepared by saturated aqueous solution-ultrasonic method. Briefly, HP-βCD and puerarin were mixed at a stoichiometric ratio of 4:1, respectively. Puerarin was accurately weighed and dissolved in absolute ethanol, and HP-βCD was dissolved in water using a water bath. Then, puerarin solution was slowly added to HP-βCD solution, and the obtained mixture was sonicated for 30 min and heated in a water bath to remove the solvent. Finally, the obtained sample was dried in a vacuum dryer at 40 °C for 24 h to obtain HP-βCD-puerarin inclusion complexes (*PIC*).

### 4.3. Fabrication of SA-g-AMPS Hydrogels

**Figure 12:**
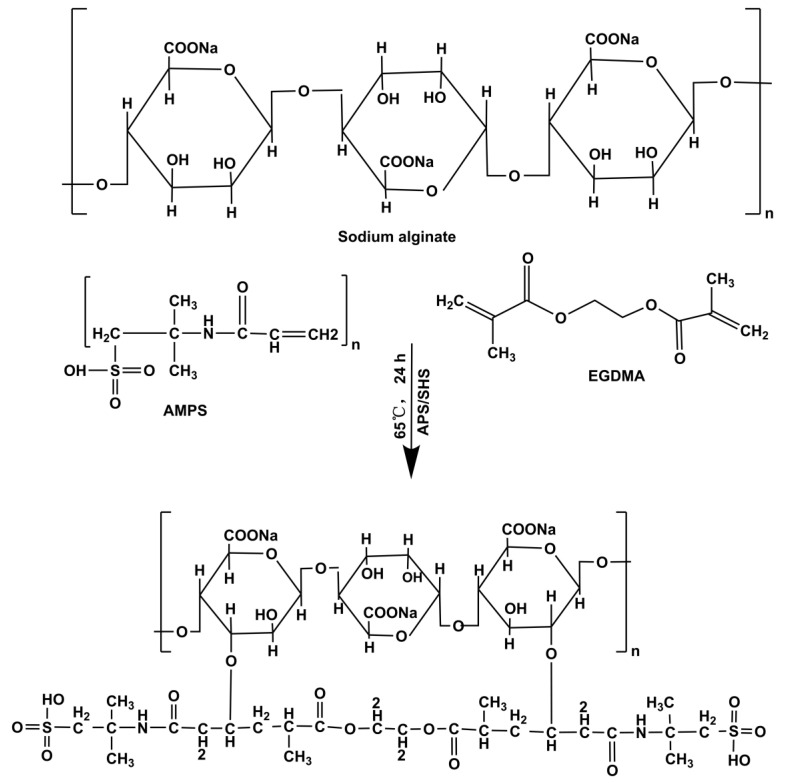
The proposed chemical structure of the synthesized SA-*g*-AMPS hydrogels.

Hydrogels were formed in several batches using free radical polymerization with minor adjustments made by linking monomers to polymeric chains [60]. Sodium alginate, APS/SHS, AMPS, and EGDMA were all precisely weighed and then placed into marked glass vials. The specified amount of water was added to each designated vial. Sodium alginate was dissolved by continuous stirring without forming lumps or precipitates. We used APS and SHS as initiators and co-initiators. SHS was dissolved in distilled water, and APS was slowly added to the solution. In addition, AMPS aqueous solution was prepared by stirring continuously at ambient temperature. The monomer solution was added drop-by-drop to the initiator/co-initiator solution while mixing continuously. AMPS and initiator/co-initiator mixtures were incorporated drop-by-drop into the sodium alginate solution by stirring and thoroughly mixing. A slow and thorough addition of EGDMA was carried out to ensure proper mixing. Finally, an appropriate amount of water was added to the reaction mixture and thoroughly mixed. Afterward, the mixture was placed in an ultrasonic bath while nitrogen bubbles were used to remove residual air. Afterward, the clear solution was covered with aluminum foil. The samples were then heated in a preheated water bath for 1 h at 50 °C, followed by an overnight increase to 65 °C. After 24 h, hydrogels were formed. Afterward, the hydrogel was cooled to room temperature in the glass molds. Hydrogels were removed from molds and cut into discs of 8 mm diameter. Discs were washed in 50:50 mixtures of ethanol and water before being transferred to Petri dishes. After one week of storage at 40 °C, the weight of the hydrogel becomes constant. Sodium alginate-g-AMPS hydrogels depicted in Table 3 contain various amounts of polymer, monomer, and cross-linker. The sodium alginate-g-AMPS hydrogels are illustrated in Figure 12.

### 4.4. PIC Loading in Hydrogels

*PIC* was incorporated into the hydrogels using the swelling-diffusion technique [61]. Specifically, a 1% solution of *PIC* was prepared by dissolving the drug in a pH 7.4 buffer, followed by the addition of preweighed, dried hydrogel, which was stirred for three days. The amount of drug loaded within the hydrogel was calculated by subtracting the weight of the unloaded hydrogel from its final weight. Moreover, the drug loading was validated by extracting a predetermined amount of drug-loaded hydrogel with buffer and detecting the concentration of puerarin at 250 nm wavelength with UV-spectrometry.

(1) $$\begin{array}{c} \mathrm{Drug}\;\mathrm{loading}=\mathrm{Drug}\;\mathrm{loaded}\;\mathrm{hydrogel}-\mathrm{Unloaded}\;\mathrm{hydrogel} \end{array}$$

### 4.5. In Vitro Characterization

#### 4.5.1. ^1^H NMR and Fourier Transform Infrared Spectroscopy (FTIR)

The ^1^H NMR spectra were acquired at 25 °C utilizing D_2_O as the solvent and trimethyl silane (TMS) as the internal standard on a Bruker AV 500 MHz (Bruker BioSpin, Zurich, Switzerland) instrument. Mnova software version 14.2.1 (Mestrelab Research, Santiago de Compostela, Spain) was used to process the data. Spectrum Two FTIR spectrometer (Perkin Elmer, Buckinghamshire, UK) with diamond attenuated total reflectance (ATR) crystal (with 16 scans) was used to assess drug-formulation interactions. Spectra were collected from 400 to 4000 cm^−1^, including both loaded and unloaded formulations, as well as pure forms of the ingredients [62].

#### 4.5.2. Thermal Study (TGA and DSC)

A thermogravimetric analyzer (Exstar TG/DTA6300TG, SII Nano, Tokyo, Japan) and differential scanning calorimetry (Perkin Elmer, Buckinghamshire, UK) were used to investigate the thermal stability of the sample [63]. Temperature-dependent weight change was assessed using a thermogravimetric analyzer. The weight profile was initially calibrated using reference standards. AMPS, *PIC*, puerarin, HP-βCD, and the formulation (0.5 to 5 mg) were placed in aluminum pans. The weight loss percentage was determined by gradually increasing the temperature by 10 °C/min with a flow rate of 10 mL/min of inert nitrogen. Differential scanning calorimetry (DSC) measurements were conducted on sodium alginate, AMPS, *PIC*, puerarin, HP-βCD, and the formulated material. The heat capacity of calorimeters was calibrated using sapphire standards. Temperature and cell constant were determined using indium as a reference [64].

#### 4.5.3. X-ray Diffraction (XRD)

XRD patterns of the pure materials and hydrogels were measured on an X-ray diffractometer (TD-3500 X-ray diffractometer, Shenzhen, China) using CuKα characteristic radiation at a voltage of 30 kV and a current of 20 mA [65]. Scanning was performed at a rate of 2°/min, and the scanning range of 2θ was 10° to 60° at room temperature. The crystallinity of a material can be determined by its X-ray diffraction peaks. Generally, sharp peaks indicate the crystallinity of pure materials, while diffuse peaks imply that the material is amorphous.

#### 4.5.4. Scanning Electron Microscopy (SEM)

A scanning electron microscope (Quanta 250 FEI, Brno-Královo Pole, Czech Republic) was used to examine the hydrogel’s structural characteristics and porosity. The method involved mounting vacuum-dried samples onto an aluminum stub and coating them with gold by sputtering. The cross-sectional morphology of the surface was thus examined under 15 kV of accelerated current [66].

#### 4.5.5. Mechanical Properties Determination

Mechanical properties, such as tensile strengths (TS, N/m^2^), and elongation at break (EAB, %), were determined for each formulation by a TA.XT texture analyzer (Stable Micro Systems, Godalming, UK). The apparatus consisted of a stainless steel spherical probe (P/5S) operating at a speed of 1.0 mm/s. TS and EAB were calculated based on the force and displacement applied by the probe in puncturing the hydrogel samples [67].

(2) $$TS=\frac{Fm}{Th}$$

(3) $$EAB=\frac{\sqrt{D^{2}+R^{2}}}{R}-1$$

where Fm refers to the maximum force that can be applied to the hydrogel by the probe, and Th is its thickness. The displacement of the probe over a period of time, from initial contact with the hydrogel to the time when the hydrogel is broken, is D, and the radius of the orifice plate is R.

#### 4.5.6. Sol–Gel Study

All formulations were subjected to a sol-gel analysis in order to determine the percentage of soluble and insoluble crosslinking portions in the hydrogels. Sol is the soluble part of the hydrogel, while the gel is the insoluble part. A Soxhlet extraction method was used to analyze the sol-gel portion of hydrogels [68]. Briefly, the preweighed hydrogel discs were placed in a flask filled with deionized distilled water. Afterward, a condenser was attached to the round-bottom flask, and the extraction process was continued for 13 h at 85 °C. The hydrogel discs were removed and placed in a vacuum oven for storage. It was weighed again after it had been completely dried. Sol-gel analysis was determined utilizing the following equations.

(4) $$Sol\;fraction\%=\frac{Z_{1}-Z_{2}}{Z_{2}}\times 100$$

(5) Gel fraction=100−Sol fraction

Z_1_ denotes the initial hydrogel weight (before extraction), and Z_2_ denotes the final hydrogel weight (after extraction).

#### 4.5.7. Porosity Determination

The porosity of all formulations of the hydrogels was evaluated and analyzed using a solvent replacement technique [69]. Dry hydrogel discs (Q1) were immersed in absolute ethanol (purity > 99.9%) for 4 days and then removed from the solvent, blotted with filter paper to remove excess solvent, and reweighed (Q2). In the same way, the disc diameters and thicknesses were measured. Porosity was calculated using the given equation.

(6) $$Porosity\;percentage\left(\%\right)=\frac{Q2-Q1}{Ρv}\times 100$$

ρ denotes ethanol density, whereas V indicates hydrogel volume (after swelling).

#### 4.5.8. Biodegradation Assessment

The biodegradation of sodium alginate-*g*-AMPS hydrogels was investigated in buffer solution pH 7.4 at 37 ± 0.5 °C [70]. Hydrogel disc weight was determined by placing them in buffer solutions pH 7.4 at different time intervals (e.g., 1, 2, 3, 4, 5, 6, and 7 days). The hydrogel discs were removed from the vacuum oven after the indicated time and dehydrated at 40 °C. The discs were then weighed again before being placed in a buffer solution of pH 7.4. Hydrogel degradation can be calculated using the given equation.

(7) $$D=\frac{p1-p2}{p1}$$

D indicates the degradation of the sample. p1 represents the weight of the dry sample, whereas p2 indicates the weight of the sample after immersion at the time (t).

### 4.6. Evaluation of Polymer Network Parameters of Hydrogels

The main parameters used to determine the structure of hydrogels in their swollen state are volume fraction (V2,s), molecular weight among crosslinked points (Mc), solvent interaction parameter (χ), and the number of crosslinks (N) [71].

#### 4.6.1. Diffusion Coefficient

Diffusion coefficients are influenced by segmental mobility and polymer properties. The diffusion coefficient was estimated using the following formula.

(8) $$D=\pi {\left(\frac{h.\theta }{4.qeq}\right)}^{2}$$

where qeq indicates equilibrium swelling of a hydrogel disc, θ indicates the slope of the linear swelling curve, and h is the thickness of the hydrogel disc.

#### 4.6.2. Volume Fraction of Polymer (V2,s)

Polymer volume fractions were calculated using the following formula.

(9) $$V2,s=\frac{1}{Veq}$$

where Veq is the volume of the hydrogel in equilibrium swollen state.

#### 4.6.3. Average Molecular Weight between Crosslinks (Mc)

Mc was obtained using the following formula.

(10) $$Mc=\frac{dpVs\left(V^{\frac{1}{3}}2,s-V2,\frac{s}{2}\right)}{\ln\left(1-V2,s\right)+V2,s+\chi V^{2}2,s}$$

where dp is the density of the polymer, and ds is the density of the solvent. vs represents the solvent’s molar volume, and χ represents the Flory–Huggins parameters that influence polymer-solvent interaction.

#### 4.6.4. Solvent Interaction Parameters (χ)

It was calculated using Flory Huggins theory.

(11) $$\chi =\frac{\ln\left(1-V2,s\right)+V2,s}{V^{2}2,s}$$

where V2,s represents the volume fraction of swollen gel at equilibrium.

#### 4.6.5. Number of Repeating Units between Crosslinks (N)

N was calculated using the data of Mc. The following formula was used to determine how many repeating units were between each crosslink.

(12) $$N=\frac{2Mc}{Mr}$$

Mr refers to the repeating unit’s molar mass. The following equation can be used to calculate it:

(13) $$Mr=\frac{mSAMSA+mAMPSMAMPS+mEGDMAMEGDMA}{mSA+mAMPS+mEGDMA}$$

where mSA, mAMPS, and mEGDMA are the masses of sodium alginate, 2-acrylamido -2-methyl-1-propanesulfonic acid, and ethylene glycol dimethacrylate, respectively. MSA, MAMPS, and MEGDMA are the molar masses of sodium alginate, 2-acrylamido -2-methyl-1-propanesulfonic acid, and ethylene glycol dimethacrylate, respectively.

### 4.7. Swelling Ratio

The swelling behavior of hydrogels was investigated at different pH values [72]. The initial weights of the hydrogel discs were recorded in dry form. The discs were then placed in simulated gastric and intestinal fluids (pH 1.2 and 7.4). The weight of the hydrogel discs was measured at specific points until a constant weight was achieved. The swelling ratio was calculated using the formula given below.

(14) $$ESR=\frac{Ws-Wd}{Wd}\times 100$$

The Ws represents the mass of the swollen hydrogel at a particular time interval, whereas the Wd represents the weight of the dried hydrogel.

### 4.8. Drug Release and Kinetic Release Data Modeling

The release of the drug from the developed hydrogel formulations was studied at pH 1.2 and 7.4 [73]. The dry discs of the drug-containing hydrogels were placed in 900 mL of phosphate buffer solution within a USP dissolution device type II at 37 ± 0.5 °C at 50 rpm. At a predetermined interval after each 5 mL sample, fresh medium of the exact same volume was added. The samples were filtered, and the UV-Vis spectrophotometer was used to examine them in triplicate at a wavelength of 250 nm.

(15) $$Drug\;release\%=\frac{\left(Amount\;of\;released\;drug\right)}{\left(Amount\;of\;loaded\;drug\right)}\times 100$$

There are many factors that can affect drug release from hydrogels, such as the relaxation of polymer chains or the hydrogel’s ability to swell, the drug’s nature, and the pH of the release medium. The controlled release process requires solvent diffusion to cause hydrogels to swell. The drug release pattern was determined using several models, including Zero order, First order, Higuchi, and Korsmeyer Peppas models.

### 4.9. Antioxidant Assays

#### 4.9.1. DPPH Activity

The antioxidant properties of synthesized hydrogels were evaluated using DPPH radical scavenging analyses [74]. A predetermined weight of samples was placed in methanol for 24 h at room temperature, in the dark. The mixture was then added to 1 mL of DPPH-methanol solution (0.1 mM). The mixture was stirred well and left to incubate for 30 min under dark conditions. The DPPH scavenging activities were then measured using a UV-Vis spectrophotometer measuring the absorbance at 517 nm. The following equation was used in the calculation.

(16) $$DPPH\left(\%\right)=\frac{A0-A}{A0}\times 100$$

while A0 indicates the absorbance of the reference sample and A refers to the absorbance of the test sample.

#### 4.9.2. ABTS Activity

The ABTS assay was utilized to test the ability of the *PIC*-loaded hydrogels to scavenge free radicals [75]. In order to cause the radicalization of the ABTS molecules, a 1:1 mixture of 7.4 mM ABTS and 2.4 mM potassium persulfate was left at room temperature overnight. The hydrogels were incubated at 37 °C in ABTS solution for 30 min. Then, the sample’s absorbance was measured at a wavelength of 730 nm. The following formula was used to calculate the radical-scavenging efficiency of ABTS [76].

(17) $$ABTS\;scavenging\;effect\left(\%\right)=\frac{A0-A1}{A0}\times 100$$

A0 corresponds to the absorbance of ABTS, while A1 corresponds to the absorbance of the samples.

### 4.10. Antibacterial Effects

Hydrogels were tested against Gram-negative and Gram-positive bacteria using the disk diffusion method. Immediately following the preparation of the agar media, it was sterilized at 121 °C. We used sterile broth to cultivate the bacterial strain. Staphylococcus aureus, Escherichia coli, and Staphylococcus aeruginosa were grown under aseptic conditions on agar media. The bacterial strains were then transferred to Petri dishes to solidify. We divided the plates into 4 sections: a negative control, a blank hydrogel, PIC-loaded hydrogels, and a control (Cefepime, 1 mg/mL). These plates were placed in an incubator for 24 h. Zones of inhibition were calculated for each sample in order to compare them.

(18) $$Percentage\;inhibition=\frac{Zone\;of\;inhibition\;of\;test\;sample\;\left(mm\right)}{Zone\;of\;inhibition\;of\;standard\;drug\;\left(mm\right)}\times 100$$

### 4.11. Statistical Analysis

The numerical data were expressed as mean ± SD. Two-way ANOVA followed by Tukey’s posthoc test was used to determine statistical differences. P-values were calculated in order to check for a significant difference and were expressed as * *p* < 0.05, ** *p* < 0.01, and *** *p* < 0.001.

## Figures and Tables

**Table 1:** The mechanical properties and drug loading of SA-g-AMPS hydrogels.

F. Codes	Thickness (mm)	TS (N/mm)	EAB (%)	*PIC*- Loaded per 1 g Hydrogel (g)
SAE-1	1.24	0.424	34.7	0.513
SAE-2	1.32	0.675	65.1	0.472
SAE-3	1.23	1.207	76.4	0.279
SAE-4	1.47	0.701	69.8	0.437
SAE-5	1.32	0.675	65.1	0.472
SAE-6	1.52	0.403	30.4	0.601
SAE-7	1.27	0.418	36.4	0.570
SAE-8	1.32	0.675	65.1	0.472
SAE-9	1.36	0.601	61.7	0.345

**Table 2:** Flory–Huggins network parameters of SA-g-AMPS hydrogels.

F. Codes	V_2,s_	χ	M_c_	M_r_	N	D × 10^−5^ (cm^2^ s^−1^)
SAE-1	0.048	0.516	2652.173	207.000	25.624	0.053
SAE-2	0.049	0.518	1695.142	206.790	16.394	0.030
SAE-3	0.060	0.520	1147.058	206.590	11.104	0.048
SAE-4	0.027	0.509	2288.888	207.384	22.073	0.015
SAE-5	0.049	0.518	1695.142	206.790	16.394	0.030
SAE-6	0.043	0.514	2333.33	207.000	22.544	0.048
SAE-7	0.030	0.510	1610.00	206.913	15.562	0.024
SAE-8	0.049	0.518	1695.142	206.790	16.394	0.030
SAE-9	0.037	0.512	2644.44	207.084	25.539	0.010

**Table 3:** Different formulations of SA-*g*-AMPS hydrogels based on various feed compositions.

Formulations	Sodium Alginate (g)	APS/SHS (g)	AMPS (g)	EGDMA (g)
SAE-1	0.5	0.3/0.3	20	**0.5**
SAE-2	0.5	0.3/0.3	20	**1**
SAE-3	0.5	0.3/0.3	20	**1.5**
SAE-4	0.5	0.3/0.3	**12**	0.5
SAE-5	0.5	0.3/0.3	**20**	0.5
SAE-6	0.5	0.3/0.3	**28**	0.5
SAE-7	**0.3**	0.3/0.3	20	0.5
SAE-8	**0.5**	0.3/0.3	20	0.5
SAE-9	**0.7**	0.3/0.3	20	0.5

Note: Bold letters indicate an increase in the feeding amount.

## Data Availability

The data is contained within the article.

## Supplementary Materials

The following supporting information can be downloaded at: https://www.mdpi.com/article/10.3390/gels9030246/s1, Table S1: Effect of materials ratio on the release of puerarin from SA-g-AMPS hydrogels cross-linked with EGDMA.

## References

[1] Naeem A., Ming Y., Pengyi H., Jie K.Y., Yali L., Haiyan Z., Shuai X., Wenjing L., Ling W., Xia Z.M. (2022). The fate of flavonoids after oral administration: A comprehensive overview of its bioavailability. Crit. Rev. Food Sci. Nutr..

[2] Taldaev A., Terekhov R., Nikitin I., Zhevlakova A., Selivanova I. (2022). Insights into the Pharmacological Effects of Flavonoids: The Systematic Review of Computer Modeling. Int. J. Mol. Sci..

[3] Naeem A., Hu P., Yang M., Zhang J., Liu Y., Zhu W., Zheng Q. (2022). Natural Products as Anticancer Agents: Current Status and Future Perspectives. Molecules.

[4] Zhang L. (2019). Pharmacokinetics and drug delivery systems for puerarin, a bioactive flavone from traditional Chinese medicine. Drug Deliv..

[5] Hou J.-Y., Gao L.-N., Meng F.-Y., Cui Y.-L. (2014). Mucoadhesive microparticles for gastroretentive delivery: Preparation, biodistribution and targeting evaluation. Mar. Drugs.

[6] Anukunwithaya T., Poo P., Hunsakunachai N., Rodsiri R., Malaivijitnond S., Khemawoot P. (2018). Absolute oral bioavailability and disposition kinetics of puerarin in female rats. BMC Pharmacol. Toxicol..

[7] Wang D., Bu T., Li Y., He Y., Yang F., Zou L. (2022). Pharmacological Activity, Pharmacokinetics, and Clinical Research Progress of Puerarin. Antioxidants.

[8] Haimhoffer Á., Rusznyák Á., Réti-Nagy K., Vasvári G., Váradi J., Vecsernyés M., Bácskay I., Fehér P., Ujhelyi Z., Fenyvesi F. (2019). Cyclodextrins in drug delivery systems and their effects on biological barriers. Sci. Pharm..

[9] Zhang D., Lv P., Zhou C., Zhao Y., Liao X., Yang B. (2019). Cyclodextrin-based delivery systems for cancer treatment. Mater. Sci. Eng. C.

[10] Haley R.M., Gottardi R., Langer R., Mitchell M.J. (2020). Cyclodextrins in drug delivery: Applications in gene and combination therapy. Drug Deliv. Transl. Res..

[11] Ding X., Zheng M., Lu J., Zhu X. (2018). Preparation and evaluation of binary and ternary inclusion complexes of fenofibrate/hydroxypropyl-β-cyclodextrin. J. Incl. Phenom. Macrocycl. Chem..

[12] Yu C., Chen X., Zhu W., Li L., Peng M., Zhong Y., Naeem A., Zang Z., Guan Y. (2022). Synthesis of Gallic Acid-Loaded Chitosan-Grafted-2-Acrylamido-2-Methylpropane Sulfonic Acid Hydrogels for Oral Controlled Drug Delivery: In Vitro Biodegradation, Antioxidant, and Antibacterial Effects. Gels.

[13] Samadian H., Maleki H., Allahyari Z., Jaymand M. (2020). Natural polymers-based light-induced hydrogels: Promising biomaterials for biomedical applications. Coord. Chem. Rev..

[14] Zang Z., Zhao S., Yang M., Yu C., Ouyang H., Chen L., Zhu W., Liao Z.-g., Naeem A., Guan Y. (2022). Blood chemical components analysis of honeysuckle and formulation of xanthan gum/starch-based (PVA-co-AA) hydrogels for controlled release. Arab. J. Chem..

[15] Omer A.M., Ahmed M.S., El-Subruiti G.M., Khalifa R.E., Eltaweil A.S. (2021). pH-sensitive alginate/carboxymethyl chitosan/aminated chitosan microcapsules for efficient encapsulation and delivery of diclofenac sodium. Pharmaceutics.

[16] İsmail O., Gökçe Kocabay Ö. (2021). Absorption and adsorption studies of polyacrylamide/sodium alginate hydrogels. Colloid Polym. Sci..

[17] Senturk Parreidt T., Müller K., Schmid M. (2018). Alginate-based edible films and coatings for food packaging applications. Foods.

[18] El-Hag Ali A., El-Rehiem H.A.A., Hegazy E.S.A., Ghobashy M.M. (2007). Characterization and Potential Application of Electro-Active Acrylamido-2-methyl Propane Sulfonic Acid/Acrylic Acid Copolymer Prepared by Ionizing Radiation. J. Macromol. Sci. Part A Pure Appl. Chem..

[19] Wang Z., Wu J., Shi X., Song F., Gao W., Liu S. (2020). Stereocomplexation of Poly (lactic acid) and Chemical Crosslinking of Ethylene Glycol Dimethacrylate (EGDMA) Double-Crosslinked Temperature/pH Dual Responsive Hydrogels. Polymers.

[20] Bulani V.D., Kothavade P.S., Nagmoti D.M., Kundaikar H.S., Degani M.S., Juvekar A.R. (2015). Characterisation and anti-inflammatory evaluation of the inclusion complex of ellagic acid with hydroxypropyl-β-cyclodextrin. J. Incl. Phenom. Macrocycl. Chem..

[21] Zhao R., Tan T., Sandström C. (2011). NMR studies on puerarin and its interaction with beta-cyclodextrin. J. Biol. Phys..

[22] Xiao Q., Gu X., Tan S. (2014). Drying process of sodium alginate films studied by two-dimensional correlation ATR-FTIR spectroscopy. Food Chem..

[23] Pourjavadi A., Hosseinzadeh H., Mazidi R. (2005). Modified carrageenan. 4. Synthesis and swelling behavior of crosslinked κC-g-AMPS superabsorbent hydrogel with antisalt and pH-responsiveness properties. J. Appl. Polym. Sci..

[24] Duran A., Soylak M., Tuncel S.A. (2008). Poly (vinyl pyridine-poly ethylene glycol methacrylate-ethylene glycol dimethacrylate) beads for heavy metal removal. J. Hazard. Mater..

[25] Cegłowski M., Kurczewska J., Lusina A., Nazim T., Ruszkowski P. (2022). EGDMA-and TRIM-Based Microparticles Imprinted with 5-Fluorouracil for Prolonged Drug Delivery. Polymers.

[26] Misiuk W., Zalewska M. (2009). Investigation of inclusion complex of trazodone hydrochloride with hydroxypropyl-β-cyclodextrin. Carbohydr. Polym..

[27] Liu B., Zhao J., Liu Y., Zhu X., Zeng J. (2012). Physiochemical properties of the inclusion complex of puerarin and glucosyl-β-cyclodextrin. J. Agric. Food Chem..

[28] Xie J., Yang F., Shi X., Zhu X., Su W., Wang P. (2013). Improvement in solubility and bioavailability of puerarin by mechanochemical preparation. Drug Dev. Ind. Pharm..

[29] Yang L.-Q., Lan Y.-Q., Guo H., Cheng L.-Z., Fan J.-Z., Cai X., Zhang L.-M., Chen R.-F., Zhou H.-S. (2010). Ophthalmic drug-loaded N, O-carboxymethyl chitosan hydrogels: Synthesis, in vitro and in vivo evaluation. Acta Pharmacol. Sin..

[30] Bao Y., Ma J., Li N. (2011). Synthesis and swelling behaviors of sodium carboxymethyl cellulose-g-poly (AA-co-AM-co-AMPS)/MMT superabsorbent hydrogel. Carbohydr. Polym..

[31] Celebioglu A., Uyar T. (2021). Electrospun formulation of acyclovir/cyclodextrin nanofibers for fast-dissolving antiviral drug delivery. Mater. Sci. Eng. C.

[32] Rani P., Mishra S., Sen G. (2013). Microwave based synthesis of polymethyl methacrylate grafted sodium alginate: Its application as flocculant. Carbohydr. Polym..

[33] Kulkarni R.V., Boppana R., Mohan G.K., Mutalik S., Kalyane N.V. (2012). pH-responsive interpenetrating network hydrogel beads of poly (acrylamide)-g-carrageenan and sodium alginate for intestinal targeted drug delivery: Synthesis, in vitro and in vivo evaluation. J. Colloid Interface Sci..

[34] Ashames A., Ullah K., Al-Tabakha M., Khan S.A., Hassan N., Mannan A., Ikram M., Buabeid M., Murtaza G. (2022). Development, characterization and In-vitro evaluation of guar gum based new polymeric matrices for controlled delivery using metformin HCl as model drug. PLoS ONE.

[35] Shaari N., Kamarudin S. (2020). Sodium alginate/alumina composite biomembrane preparation and performance in DMFC application. Polym. Test..

[36] Hassani A., Mahmood S., Enezei H.H., Hussain S.A., Hamad H.A., Aldoghachi A.F., Hagar A., Doolaanea A.A., Ibrahim W.N. (2020). Formulation, characterization and biological activity screening of sodium alginate-gum arabic nanoparticles loaded with curcumin. Molecules.

[37] Li P., Jia H., Zhang S., Yang Y., Sun H., Wang H., Pan W., Yin F., Yang X. (2020). Thermal extrusion 3D printing for the fabrication of puerarin immediate-release tablets. AAPS PharmSciTech.

[38] Zheng L., Xu H., Hu H., Ruan J., Shi C., Cao J., Zhang X. (2022). Preparation, characterization and antioxidant activity of inclusion complex loaded with puerarin and corn peptide. Food Biosci..

[39] Khushbu, Jindal R. (2021). Comparative evaluation for controlled release of amoxicillin from RSM-CCD-optimized nanocomposites based on sodium alginate and chitosan-containing inclusion complexes. Mol. Pharm..

[40] Cheng M., Yuan F., Liu J., Liu W., Feng J., Jin Y., Tu L. (2020). Fabrication of fine puerarin nanocrystals by Box–Behnken Design to enhance intestinal absorption. Aaps Pharmscitech.

[41] Liu Y., Chen Y., Gao X., Fu J., Hu L. (2022). Application of cyclodextrin in food industry. Crit. Rev. Food Sci. Nutr..

[42] Khan M.U.A., Yaqoob Z., Ansari M.N.M., Razak S.I.A., Raza M.A., Sajjad A., Haider S., Busra F.M. (2021). Chitosan/poly vinyl alcohol/graphene oxide based pH-responsive composite hydrogel films: Drug release, anti-microbial and cell viability studies. Polymers.

[43] Park D.W., Haam S., Lee T.G., Kim H.S., Kim W.S. (2004). Chemoenzymatic synthesis of sugar-containing biocompatible hydrogels: Crosslinked poly (β-methylglucoside acrylate) and poly (β-methylglucoside methacrylate). J. Biomed. Mater. Res. Part A Off. J. Soc. Biomater. Jpn. Soc. Biomater. Aust. Soc. Biomater. Korean Soc. Biomater..

[44] Chen C., Peng Z., Gu J., Peng Y., Huang X., Wu L. (2020). Exploring environmentally friendly biopolymer material effect on soil tensile and compressive behavior. Int. J. Environ. Res. Public Health.

[45] Khan Z., Minhas M.U., Ahmad M., Khan K.U., Sohail M., Khalid I. (2020). Functionalized pectin hydrogels by cross-linking with monomer: Synthesis, characterization, drug release and pectinase degradation studies. Polym. Bull..

[46] Chatterjee S., Hui P.C.-l. (2021). Review of applications and future prospects of stimuli-responsive hydrogel based on thermo-responsive biopolymers in drug delivery systems. Polymers.

[47] Dragan E.S., Dinu M.V. (2020). Advances in porous chitosan-based composite hydrogels: Synthesis and applications. React. Funct. Polym..

[48] Khan S.A., Azam W., Ashames A., Fahelelbom K.M., Ullah K., Mannan A., Murtaza G. (2020). β-Cyclodextrin-based (IA-co-AMPS) Semi-IPNs as smart biomaterials for oral delivery of hydrophilic drugs: Synthesis, characterization, in-Vitro and in-Vivo evaluation. J. Drug Deliv. Sci. Technol..

[49] Ghauri Z.H., Islam A., Qadir M.A., Gull N., Haider B., Khan R.U., Riaz T. (2021). Development and evaluation of pH-sensitive biodegradable ternary blended hydrogel films (Chitosan/Guar gum/PVP) for drug delivery application. Sci. Rep..

[50] Dragan E.S. (2014). Design and applications of interpenetrating polymer network hydrogels. A review. Chem. Eng. J..

[51] Butt A., Jabeen S., Nisar N., Islam A., Gull N., Iqbal S.S., Khan S.M., Yameen B. (2019). Controlled release of cephradine by biopolymers based target specific crosslinked hydrogels. Int. J. Biol. Macromol..

[52] Karoyo A.H., Wilson L.D. (2021). A review on the design and hydration properties of natural polymer-based hydrogels. Materials.

[53] Abbasi A.R., Sohail M., Minhas M.U., Khaliq T., Kousar M., Khan S., Hussain Z., Munir A. (2020). Bioinspired sodium alginate based thermosensitive hydrogel membranes for accelerated wound healing. Int. J. Biol. Macromol..

[54] Abd El-Ghaffar M., Hashem M., El-Awady M., Rabie A. (2012). pH-sensitive sodium alginate hydrogels for riboflavin controlled release. Carbohydr. Polym..

[55] Khalid I., Ahmad M., Minhas M.U., Barkat K. (2018). Synthesis and evaluation of chondroitin sulfate based hydrogels of loxoprofen with adjustable properties as controlled release carriers. Carbohydr. Polym..

[56] Anwar H., Ahmad M., Minhas M.U., Rehmani S. (2017). Alginate-polyvinyl alcohol based interpenetrating polymer network for prolonged drug therapy, optimization and in-vitro characterization. Carbohydr. Polym..

[57] Hanna D.H., Lotfy V.F., Basta A.H., Saad G.R. (2020). Comparative evaluation for controlling release of niacin from protein-and cellulose-chitosan based hydrogels. Int. J. Biol. Macromol..

[58] Yang Y., Ye H., Zhao C., Ren L., Wang C., Georgiev M.I., Xiao J., Zhang T. (2021). Value added immunoregulatory polysaccharides of Hericium erinaceus and their effect on the gut microbiota. Carbohydr. Polym..

[59] Malik N.S., Ahmad M., Minhas M.U., Tulain R., Barkat K., Khalid I., Khalid Q. (2020). Chitosan/xanthan gum based hydrogels as potential carrier for an antiviral drug: Fabrication, characterization, and safety evaluation. Front. Chem..

[60] Guan Y., Yu C., Zang Z., Wan X., Naeem A., Zhang R., Zhu W. (2022). Chitosan/xanthan gum-based (Hydroxypropyl methylcellulose-co-2-Acrylamido-2-methylpropane sulfonic acid) interpenetrating hydrogels for controlled release of amorphous solid dispersion of bioactive constituents of Pueraria lobatae. Int. J. Biol. Macromol..

[61] Naeem A., Yu C., Zhu W., Chen X., Wu X., Chen L., Zang Z., Guan Y. (2022). Gallic Acid-Loaded Sodium Alginate-Based (Polyvinyl Alcohol-Co-Acrylic Acid) Hydrogel Membranes for Cutaneous Wound Healing: Synthesis and Characterization. Molecules.

[62] Karnakar R.R., Gite V.V. (2022). Eco-friendly slow release of ZnSO4 as a micronutrient from poly (acrylic acid: Acrylamide) and guar gum based crosslinked biodegradable hydrogels. Polym.-Plast. Technol. Mater..

[63] Ghadami A., Taheri S., Alinejad Z., Dinari M. (2022). Preparation of acrylate-based double and triple interpenetrating polymer networks hydrogels: Rheological, thermal, and swelling behavior. Polym. Adv. Technol..

[64] Sulaeman A.S., Putro P.A., Nikmatin S. (2022). Thermal studies of hydrogels based on poly (acrylic acid) and its copolymers by differential scanning calorimetry: A systematic literature review. Polym. Polym. Compos..

[65] Abdalla T.H., Nasr A.S., Bassioni G., Harding D.R., Kandile N.G. (2022). Fabrication of sustainable hydrogels-based chitosan Schiff base and their potential applications. Arab. J. Chem..

[66] Kocak F.Z., Yar M., Rehman I.U. (2022). Hydroxyapatite-integrated, heparin-and glycerol-functionalized chitosan-based injectable hydrogels with improved mechanical and proangiogenic performance. Int. J. Mol. Sci..

[67] Du M., Zhang Y., Zhao Y., Fang Y. (2023). Agarose/konjac glucomannan double network hydrogels to mimic the texture of beef tripe. Food Hydrocoll..

[68] Siddiqua A., Ranjha N.M., Rehman S., Shoukat H., Ramzan N., Sultana H. (2022). Preparation and characterization of methylene bisacrylamide crosslinked pectin/acrylamide hydrogels. Polym. Bull..

[69] Araújo D., Rodrigues T., Alves V.D., Freitas F. (2022). Chitin-glucan complex hydrogels: Optimization of gel formation and demonstration of drug loading and release ability. Polymers.

[70] Azeem M.K., Islam A., Rizwan M., Rasool A., Gul N., Khan R.U., Khan S.M., Rasheed T. (2023). Sustainable and environment Friendlier carrageenan-based pH-responsive hydrogels: Swelling behavior and controlled release of fertilizers. Colloid Polym. Sci..

[71] Singh B., Sharma V. (2014). Influence of polymer network parameters of tragacanth gum-based pH responsive hydrogels on drug delivery. Carbohydr. Polym..

[72] Khan M.U.A., Abd Razak S.I., Haider S., Mannan H.A., Hussain J., Hasan A. (2022). Sodium alginate-f-GO composite hydrogels for tissue regeneration and antitumor applications. Int. J. Biol. Macromol..

[73] Liu Y., Ran Y., Ge Y., Raza F., Li S., Zafar H., Wu Y., Paiva-Santos A.C., Yu C., Sun M. (2022). pH-sensitive peptide hydrogels as a combination drug delivery system for cancer treatment. Pharmaceutics.

[74] Muangsri R., Chuysinuan P., Thanyacharoen T., Techasakul S., Sukhavattanakul P., Ummartyotin S. (2022). Release Characteristic and Antioxidant Activity of 4-Hydroxybenzoic Acid (4HB) from Sodium Alginate and Polyvinyl Alcohol-based Hydrogel. ChemistrySelect.

[75] Tunit P., Thammarat P., Okonogi S., Chittasupho C. (2022). Hydrogel Containing Borassus flabellifer L. Male Flower Extract for Antioxidant, Antimicrobial, and Anti-Inflammatory Activity. Gels.

[76] Lorz L.R., Yoo B.C., Kim M.-Y., Cho J.Y. (2019). Anti-wrinkling and anti-melanogenic effect of Pradosia mutisii methanol extract. Int. J. Mol. Sci..

