# Supplementary material for: Study of Hydroxypropyl β-Cyclodextrin and Puerarin Inclusion Complexes Encapsulated in Sodium Alginate-Grafted 2-Acrylamido-2-Methyl-1-Propane Sulfonic Acid Hydrogels for Oral Controlled Drug Delivery
Source: Gels. 2023 Mar 20;9(3):246. doi: 10.3390/gels9030246 (PMC10048200; doi:10.3390/gels9030246)
Supplement: Supplementary file 1 [file gels-09-00246-s001.zip › gels-2264930-supplementary.pdf]

Supplementary Materials

**Study of Hydroxypropyl β-Cyclodextrin and Puerarin Inclusion Complexes Encapsulated in Sodium Alginate-Grafted-2-Acrylamido -2-Methyl-1-Propane Sulfonic Acid Hydrogels for Oral Controlled Drug Delivery**

**Table S1.** Effect of materials ratio on the release of puerarin from SA-g-AMPS hydrogels cross-linked with EGDMA.

| F. Codes | pH | Zero Order | | First Order | | Higuchi Model | | Korsmeyer-Peppas Model | |
|---|---|---|---|---|---|---|---|---|---|
| | | $K_0$ ($h^{-1}$) | $r^2$ | $K_1$ ($h^{-1}$) | $r^2$ | $K_2$ ($h^{-1}$) | $r^2$ | $r^2$ | n |
| **SAE-1** | 1.2 | 1.278 | 0.9732 | 0.018 | 0.9899 | 7.242 | 0.9987 | 0.9991 | 0.519 |
| | 7.4 | 0.933 | 0.9694 | 0.012 | 0.9817 | 5.325 | 0.9993 | 0.9988 | 0.473 |
| SAE-2 | 1.2 | 1.056 | 0.9561 | 0.014 | 0.9747 | 0.038 | 0.9970 | 0.9975 | 0.457 |
| | 7.4 | 1.049 | 0.9640 | 0.014 | 0.9800 | 0.009 | 0.9994 | 0.9990 | 0.457 |
| SAE-3 | 1.2 | 0.802 | 0.9553 | 0.010 | 0.9684 | 4.611 | 0.9970 | 0.9970 | 0.434 |
| | 7.4 | 0.688 | 0.9217 | 0.008 | 0.9361 | 4.051 | 0.9893 | 0.9974 | 0.320 |
| SAE-4 | 1.2 | 1.218 | 0.9712 | 0.016 | 0.9877 | 0.907 | 0.9976 | 0.9981 | 0.519 |
| | 7.4 | 0.901 | 0.9572 | 0.011 | 0.9706 | 5.189 | 0.9966 | 0.9964 | 0.424 |
| SAE-5 | 1.2 | 1.056 | 0.9561 | 0.014 | 0.9747 | 0.038 | 0.9970 | 0.9975 | 0.457 |
| | 7.4 | 1.049 | 0.9640 | 0.014 | 0.9800 | 0.009 | 0.9994 | 0.9990 | 0.457 |
| SAE-6 | 1.2 | 1.258 | 0..9479 | 0.017 | 0.9741 | 7.188 | 0.9938 | 0.9940 | 0.488 |
| | 7.4 | 0.774 | 0.9516 | 0.009 | 0.9641 | 4.466 | 0.9968 | 0.9978 | 0.417 |
| SAE-7 | 1.2 | 1.285 | 0.9664 | 0.018 | 0.9857 | 7.308 | 0.9990 | 0.9990 | 0.503 |
| | 7.4 | 0.850 | 0.9627 | 0.011 | 0.9986 | 4.870 | 0.9985 | 0.9981 | 0.449 |
| SAE-8 | 1.2 | 1.056 | 0.9561 | 0.014 | 0.9747 | 0.038 | 0.9970 | 0.9975 | 0.457 |

| | | | | | | | | | |
|---|---|---|---|---|---|---|---|---|---|
| | 7.4 | 1.049 | 0.9640 | 0.014 | 0.9800 | 0.009 | 0.9994 | 0.9990 | 0.457 |
| SAE-9 | 1.2 | 0.955 | 0.9499 | 0.012 | 0.9684 | 5.484 | 0.9963 | 0.9968 | 0.453 |
| | 7.4 | 0.879 | 0.9565 | 0.011 | 0.9709 | 5.058 | 0.9983 | 0.9983 | 0.438 |
